# Supplementary material for: Diversity and asynchrony in soil microbial communities stabilizes ecosystem functioning
Source: eLife. 2021 Mar 23;10:e62813. doi: 10.7554/eLife.62813 (PMC7987343; doi:10.7554/eLife.62813)
Supplement: Supplementary file 1. [file elife-62813-supp1.docx]

**Supplementary file 1**

Results of ANOVA models assessing the effects of soil biodiversity treatments on microbial richness, composition and ecosystem functions and effect of microbial richness, asynchrony and stability in the abundance of microbes on the stability of ecosystem functions.

**Supplementary file 1 – table 1.** Mixed effect ANOVA results for the variation in fungal and bacterial richness. ‘Sieve log’ is the log of the sieve size used to create our soil biodiversity treatment gradient and is a continuous variable to assess the expected decline in microbial richness along our soil biodiversity treatment gradient. ‘Sieve factor’ captures the deviation away from a linear relationship. ‘Time’ linear assess temporal trends followed by time as a factor to indicate where temporal changes are non-linear. The indented terms in italics indicate the results of including a ‘*Sterile*’ term as a contrast term accounting for our sterile treatment driving significant effects of the biodiversity treatment gradient. The terms in bold are model results prior to the inclusion of the ‘*Sterile*’ contrast term. The residual effect of the sieving gradient after accounting for the effect of the sterile soil is indicated by *Resid*. Random terms include ‘Field’, which is the field location from which the soil inoculum originated that were organized within blocks within the greenhouse and ‘Microcosm’ is the identity of the individual experimental unit.

|  |  | Richness (ITS) | | | Richness (16S) | | |
| --- | --- | --- | --- | --- | --- | --- | --- |
| Fixed | *DF_n_* | *DF_d_* | *F* | *P* | *DF_d_* | *F* | *P* |
| *Sterile (Str)* | *1* | *46.4* | *157.80* | *<0.001* | 46.0 | 838.30 | *<0.001* |
| *Resid. S_log_* | *1* | *43.6* | *14.96* | *<0.001* | 43.5 | 1.40 | 0.249 |
| *Resid. S_fac_* | *1* | *41.7* | *0.93* | *0.340* | 41.3 | 0.01 | 0.936 |
| **Sieve log (S_log_)** | **1** | **46.0** | **172.00** | **<0.001** | **45.7** | **753.90** | ***<0.001*** |
| **Sieve factor (S_fac_)** | **2** | **42.9** | **0.83** | **0.445** | **42.5** | **42.90** | ***<0.001*** |
| **Time linear (T_lin_)** | **1** | **104.0** | **0.21** | **0.644** | **104.7** | **35.5** | ***<0.001*** |
| **Time factor (T_fac_)** | **3** | **166.8** | **1.75** | **0.159** | **178.7** | **3.80** | **0.011** |
| **T_fac_ × S_log_** | **4** | **170.1** | **0.75** | **0.561** | **177.1** | **1.6** | **0.174** |
| *Str × T_lin_* | *1* | *104* | *15.63* | *<0.001* |  |  |  |
| *Resid. T_lin_ × S_fac_* | *1* | *104* | *12.16* | *<0.001* |  |  |  |
| **T_lin_ × S_fac_** | **2** | **104** | **13.9** | **<0.001** | **104.7** | **2.3** | **0.101** |
|  |  |  |  |  |  |  |  |
| Random |  | *Var* 10^2^ | *SE* 10^2^ |  |  | *Var.* 10^3^ | *SE* 10^3^ |
| Microcosm |  | 3.47 | 2.07 |  |  | -8.37 | 10.71 |
| Field in Block |  | -0.75 | 0.74 |  |  | 0.21 | 3.73 |
| Residual |  | 30.17 | 2.86 |  |  | 213.24 | 21.01 |
| Time ρ_AR1_ |  | -0.106 | 0.086 |  |  | -0.031 | 0.082 |

*DF_n_* = numerator degrees of freedom, *DF_n_* = denominator degrees of freedom, *F* = variance ratio, *P* = probability of type I error, *Var.* = variance component, *SE* = associated standard error. Time ρ_AR1_ is the AR1 autocorrelation through time.

**Supplementary file 1 – table 2.** Summary of PERMANOVA results for the changes in the composition of the fungal and bacterial communities. **a**) provides results on the community compositional dissimilarities among our soil biodiversity treatments and time points. The effect of the sterile soil treatment (Sterile) is partitioned out as a linear contrast term of Treatment. The field from which the soil communities originated, and the block are included as covariates. **b**) Summary of the same PERMANOVA model for each time point separately.

|  |  | Fungi | | | Bacteria | | |
| --- | --- | --- | --- | --- | --- | --- | --- |
| **a)** | *Df* | *R^2^* | *F* | *P* | *R^2^* | *F* | *P* |
| Block | 7 | 0.047 | 2.18 | 0.001 | 0.049 | 2.41 | <0.001 |
| Field | 2 | 0.025 | 3.98 | 0.001 | 0.029 | 4.88 | <0.001 |
| Sterile (Str) | 1 | 0.016 | 5.04 | 0.001 | 0.037 | 12.71 | <0.001 |
| Treatment (Trt) | 2 | 0.018 | 2.83 | 0.001 | 0.011 | 1.93 | <0.001 |
| Time (T) | 4 | 0.018 | 1.48 | 0.001 | 0.038 | 3.25 | <0.001 |
| T × Str | 4 | 0.008 | 0.67 | 1.000 | 0.016 | 1.38 | <0.001 |
| T × Trt | 8 | 0.032 | 1.30 | 0.001 | 0.023 | 0.99 | 0.522 |
| Residual | 271 | 0.837 |  |  | 0.796 |  |  |

| **b)** |  |  | Fungi | | | | Bacteria | | | |
| --- | --- | --- | --- | --- | --- | --- | --- | --- | --- | --- |
| Time |  | *Df* | *R^2^* | *F* | *P* | *R^2^* | | *F* | *P* |  |
| 11 weeks | Sterile | 1 | 0.027 | 1.64 | 0.001 | 0.047 | | 2.85 | <0.001 |  |
|  | Treatment | 2 | 0.059 | 1.79 | 0.001 | 0.040 | | 1.23 | 0.011 |  |
| 22 weeks | Sterile | 1 | 0.025 | 1.49 | 0.003 | 0.052 | | 3.17 | <0.001 |  |
|  | Treatment | 2 | 0.045 | 1.36 | 0.001 | 0.036 | | 1.10 | 0.101 |  |
| 33 weeks | Sterile | 1 | 0.029 | 1.74 | 0.001 | 0.059 | | 3.67 | <0.001 |  |
|  | Treatment | 2 | 0.045 | 1.36 | 0.001 | 0.036 | | 1.11 | 0.058 |  |
| 44 weeks | Sterile | 1 | 0.030 | 1.81 | 0.001 | 0.060 | | 3.71 | <0.001 |  |
|  | Treatment | 2 | 0.046 | 1.39 | 0.001 | 0.036 | | 1.10 | 0.063 |  |
| 55 weeks | Sterile | 1 | 0.024 | 1.46 | 0.024 | 0.062 | | 3.89 | <0.001 |  |
|  | Treatment | 2 | 0.061 | 1.86 | 0.001 | 0.039 | | 1.20 | 0.013 |  |

**Supplementary file 1 – table 3.** Mixed effect ANOVA results for the variation in plant biomass production and diversity. ‘Sieve log’ is the log of the sieve size used to create our soil biodiversity treatment gradient and is a continuous variable to assess the expected decline in microbial richness along our soil biodiversity treatment gradient. ‘Sieve factor’ captures the deviation away from a linear relationship. ‘Time’ linear assess temporal trends followed by time as a factor to indicate where temporal changes are non-linear. The indented terms in italics indicate the results of including a ‘*Sterile*’ term as a contrast term accounting for our sterile treatment driving significant effects of the biodiversity treatment gradient. The terms in bold are model results prior to the inclusion of the ‘*Sterile*’ contrast term. The residual effect of the sieving gradient after accounting for the effect of the sterile soil is indicated by *Resid*. Random terms include ‘Field’, which is the field location from which the soil inoculum originated that were organized within blocks within the greenhouse and ‘Microcosm’ is the identity of the individual experimental unit.

|  |  | Biomass production (g) | | | Plant diversity (*H’*) | | |
| --- | --- | --- | --- | --- | --- | --- | --- |
| Fixed | *DF_n_* | *DF_d_* | *F* | *P* | *DF_d_* | *F* | *P* |
| *Sterile (Str)* | *1* | *43.1* | *26.43* | *<0.001* | *43.4* | *369.70* | *<0.001* |
| *Resid. S_log_* | *1* | *40.7* | *4.71* | *0.036* | *41.3* | *5.08* | *0.030* |
| *Resid. S_fac_* | *1* | *38* | *3.49* | *0.069* | *38.9* | *2.43* | *0.127* |
| **Sieve log (S_log_)** | **1** | **43.0** | **31.47** | **<0.001** | **43.4** | **352.70** | **<0.001** |
| **Sieve factor (S_fac_)** | **2** | **39.4** | **1.77** | **0.184** | **40.1** | **13.14** | **<0.001** |
| **Time linear (T_lin_)** | **1** | **76.6** | **4.09** | **0.047** | **78.3** | **233.50** | **<0.001** |
| **Time factor (T_fac_)** | **3** | **177** | **249.70** | **<0.001** | **188.6** | **27.11** | **<0.001** |
| *Str × T_fac_* | *4* | *170.3* | *29.77* | *<0.001* | *185* | *19.18* | *<0.001* |
| *Resid. T_fac_ × S_log_* | *4* | *170.3* | *2.69* | *0.033* | *185* | *4.44* | *0.002* |
| *Resid. T_lin_ × S_fac_* | *1* | *77.1* | *0.63* | *0.428* | *78.7* | *3.8* | *0.055* |
| **T_fac_ × S_log_** | **4** | **171.9** | **30.08** | **<0.001** | **186.6** | **16.06** | **<0.001** |
| **T_lin_ × S_fac_** | **2** | **76.6** | **3.22** | **0.045** | **78.3** | **12.72** | **<0.001** |
|  |  |  |  |  |  |  |  |
| Random |  | *Var* | *SE* |  |  | *Var.* 10^-2^ | *SE* 10^-2^ |
| Microcosm |  | -0.156 | 0.672 |  |  | -4.03 | 3.60 |
| Field in Block |  | 0.670 | 0.450 |  |  | 0.82 | 0.43 |
| Residual |  | 5.177 | 0.818 |  |  | 7.21 | 3.81 |
| Time ρ_AR1_ |  | 0.366 | 0.102 |  |  | 0.829 | 0.091 |

*DF_n_* = numerator degrees of freedom, *DF_n_* = denominator degrees of freedom, *F* = variance ratio, *P* = probability of type I error, *Var.* = variance component, *SE* = associated standard error. Time ρ_AR1_ is the AR1 autocorrelation through time.

**Supplementary file 1 – table 4.** Mixed effect ANOVA results for the variation in litter decomposition and soil carbon assimilation. ‘Sieve log’ is the log of the sieve size used to create our soil biodiversity treatment gradient and is a continuous variable to assess the expected decline in microbial richness along our soil biodiversity treatment gradient. ‘Sieve factor’ captures the deviation away from a linear relationship. ‘Time’ linear assess temporal trends followed by time as a factor to indicate where temporal changes are non-linear. The indented terms in italics indicate the results of including a ‘*Sterile*’ term as a contrast term accounting for our sterile treatment driving significant effects of the biodiversity treatment gradient. The terms in bold are model results prior to the inclusion of the ‘*Sterile*’ contrast term. The residual effect of the sieving gradient after accounting for the effect of the sterile soil is indicated by *Resid*. Random terms include ‘Field’, which is the field location from which the soil inoculum originated that were organized within blocks within the greenhouse and ‘Microcosm’ is the identity of the individual experimental unit.

|  |  | Litter decomposition (Prop.) | | | Carbon assimilation (d^13^C) | | |
| --- | --- | --- | --- | --- | --- | --- | --- |
| Fixed | *DF_n_* | *DF_d_* | *F* | *P* | *DF_d_* | *F* | *P* |
| *Sterile (Str)* | *1* | *44.8* | *10.12* | *0.003* | *41.9* | *6.43* | *0.015* |
| *Resid. S_log_* | *1* | *39.1* | *2.96* | *0.094* | *39.7* | *0.63* | *0.433* |
| *Resid. S_fac_* | *1* | *38.5* | *0.75* | *0.393* | *37.6* | *<0.01* | *0.988* |
| **Sieve log (S_log_)** | **1** | **43.7** | **12.85** | **<0.001** | **41.9** | **4.29** | **0.044** |
| **Sieve factor (S_fac_)** | **2** | **39.3** | **0.48** | **0.621** | **38.7** | **1.38** | **0.264** |
| **Time linear (T_lin_)** | **1** | **91.0** | **3.42** | **0.068** | **92.2** | **382.00** | **<0.001** |
| **Time factor (T_fac_)** | **3** | **171.5** | **23.98** | **<0.001** | **145.8** | **61.46** | **<0.001** |
| **T_fac_ × S_log_** | **4** | **170.2** | **0.84** | **0.501** | **151.3** | **2.35** | **0.057** |
| **T_lin_ × S_fac_** | **2** | **90.3** | **0.51** | **0.602** | **93.1** | **1.56** | **0.215** |
|  |  |  |  |  |  |  |  |
| Random |  | *Var.* 10^-3^ | *SE* 10^-3^ |  |  | *Var.* 10^-2^ | *SE* 10^-2^ |
| Microcosm |  | 0.79 | 1.07 |  |  | 1.33 | 0.54 |
| Field in Block |  | 0.93 | 0.76 |  |  | 2.27 | 1.14 |
| Residual |  | 11.98 | 1.35 |  |  | 4.33 | 0.46 |
| Time ρ_AR1_ |  | 0.110 | 0.091 |  |  | -0.050 | 0.101 |

*DF_n_* = numerator degrees of freedom, *DF_n_* = denominator degrees of freedom, *F* = variance ratio, *P* = probability of type I error, *Var.* = variance component, *SE* = associated standard error. Time ρ_AR1_ is the AR1 autocorrelation through time. Note, litter decomposition (proportion of litter mass loss) was squared prior to analyses to meet model assumptions of homoscedasticity.

**Supplementary file 1 - table 5.** Summary ANOVA results for the effect of bacterial and fungal richness and the microbial diversity index (their scaled average) on the stability of ecosystem functioning after first accounting for the sterile soil treatment. The effect of the sterile soil treatment was fitted ahead of richness measures and the residual effect of richness are shown in italics. For comparison the overall effect of richness prior to accounting for the sterile soil treatment is shown in the grey shaded row below. The bold results highlight where the effect of richness remained significant after accounting for the sterile soil treatment.

|  |  |  | **Bacteria** | | **Fungi** | | **Microbial diversity** | |
| --- | --- | --- | --- | --- | --- | --- | --- | --- |
|  | *DF_num_* | *DF_den_* | *F* | *P* | *F* | *P* | *F* | *P* |
| **Biomass production** | | | | | | | | |
| *Sterile treatment* | *1* | *39* | *32.28* | *<0.001* | *37.03* | *<0.001* | *37.70* | *<0.001* |
| *Richness residual* | *1* | *39* | *0.09* | *0.764* | ***4.47*** | ***0.041*** | ***4.58*** | ***0.039*** |
| Richness overall | 1 | 40 | 32.16 | <0.001 | 38.34 | <0.001 | 43.17 | <0.001 |
| **Plant Diversity** | | | | | | | | |
| *Sterile treatment* | *1* | *39* | *25.46* | *<0.001* | *24.99* | *<0.001* | *25.00* | *<0.001* |
| *Richness residual* | *1* | *39* | *1.01* | *0.320* | *<0.01* | *0.982* | *0.18* | *0.671* |
| Richness overall | 1 | 40 | 26.90 | <0.001 | 14.86 | <0.001 | 23.20 | <0.001 |
| **Decomposition** | | | | | | | | |
| *Sterile treatment* | *1* | *39* | *2.83* | *0.101* | *2.75* | *0.105* | *2.80* | *0.103* |
| *Richness residual* | *1* | *39* | *1.30* | *0.262* | *1.71* | *0.199* | *2.66* | *0.111* |
| Richness overall | 1 | 40 | 3.53 | 0.068 | 4.51 | 0.040 | 4.45 | 0.041 |
| **C accumulation** | | | | | | | | |
| *Sterile treatment* | *1* | *39* | *3.75* | *0.060* | *3.61* | *0.065* | *3.62* | *0.065* |
| *Richness residual* | *1* | *39* | *1.17* | *0.287* | *0.15* | *0.699* | *0.01* | *0.941* |
| Richness overall | 1 | 40 | 4.64 | 0.037 | 1.81 | 0.186 | 3.41 | 0.072 |
| **Multifunctional stability** |  |  |  |  |  |  |  |  |
| *Sterile treatment* | *1* | *39* | *32.48* | *<0.001* | *30.93* | *<0.001* | *32.17* | *0.065* |
| *Richness residual* | *1* | *39* | *1.35* | *0.253* | *1.02* | *0.318* | *1.89* | *0.177* |
| Richness overall | 1 | 40 | 34.71 | <0.001 | 24.44 | <0.001 | 34.48 | <0.001 |

**Supplementary file 1 - table 6.** Regression model results for the effects of soil biodiversity (sDiv) and plant diversity (pDiv) on stability in plant productivity when considered independently or simultaneously. Model fit statistics are provided: AICc, and marginal R^2^ (*R^2^_Margin_*). Standardized coefficients are provided. Significance are indicated by: * *P* < 0.05, ** *P* < 0.01 and *** *P* < 0.001

| **Temporal stability in productivity** | | | | | |
| --- | --- | --- | --- | --- | --- |
| Model | sDiv | pDiv | AICc | *R^2^_Margin_* |  |
| 1 | ***0.644 |  | -43.0 | 0.389 |  |
| 2 | ***0.660 | -0.013 | -38.3 | 0.385 |  |
| 3 |  | ***0.376 | -30.5 | 0.286 |  |

**Supplementary file 1 - table 7.** Regression model results for the effects of soil diversity (sDiv), plant diversity (pDiv) and plant productivity (BM) on stability in carbon assimilation when considered independently or simultaneously. Model fit statistics are provided: AICc, marginal R^2^ (*R^2^_Margin_*). Standardized coefficients are provided, and their significance are indicated by: * *P* < 0.05.

|  |  |  |  |  |  |  |
| --- | --- | --- | --- | --- | --- | --- |
| Model | sDiv | pDiv | BM | *CV_BM_* | AICc | *R^2^_Margin_* |
| 1 |  |  |  | *0.243 | -44.6 | 0.071 |
| 2 | 0.198 |  |  |  | -42.8 | 0.045 |
| 3 | 0.075 |  |  | 0.188 | -40.2 | 0.067 |
| 4 | *0.421 | -0.176 |  |  | -40.1 | 0.064 |
| 5 |  |  | 0.132 |  | -39.9 | 0.007 |
| 6 | *0.224 |  | -0.089 |  | -39.2 | 0.048 |
| 7 |  | 0.075 |  |  | -38.7 | 0.014 |
| 8 | *0.468 | -0.185 | -0.123 |  | -36.6 | 0.067 |
| 9 |  | 0.066 | 0.058 |  | -34.9 | 0.014 |

**Supplementary file 1 - table 8.** Summary ANOVA results for the effect of the mean stability in the abundance of individual bacterial and fungal taxa that support a given function on the stability of that ecosystem function after first accounting for the sterile soil treatment. The effect of the sterile soil treatment was fitted ahead of species stability and the residual effect of species stability are shown in italics. For comparison the overall effect of species synchrony prior to accounting for the sterile soil treatment is shown in the grey shaded row below. Results in bold highlight where the effect of species stability remained significant after accounting for the sterile soil treatment. Microbial diversity is the scaled average of the species stability of the bacterial and fungal taxa.

|  |  |  | **Bacteria** | | **Fungi** | | **Microbial diversity** | |
| --- | --- | --- | --- | --- | --- | --- | --- | --- |
|  | *DF_num_* | *DF_den_* | *F* | *P* | *F* | *P* | *F* | *P* |
| **Biomass production** | | | | | | | | |
| *Sterile treatment* | *1* | *39* | *28.24* | *<0.001* | *27.78* | *<0.001* | *27.87* | *<0.001* |
| *Species stability residual* | *1* | *39* | *0.95* | *0.335* | *0.01* | *0.930* | *0.19* | *0.661* |
| Species stability overall | 1 | 40 | 27.77 | *<0.001* | 11.80 | 0.001 | 21.51 | *<0.001* |
| **Plant Diversity** | | | | | | | | |
| *Sterile treatment* | *1* | *39* | *16.39* | *<0.001* | *16.30* | *<0.001* | *16.17* | *<0.001* |
| *Species stability residual* | *1* | *39* | *0.82* | *0.368* | *0.49* | *0.485* | *0.05* | *0.822* |
| Species stability overall | 1 | 40 | 17.04 | *<0.001* | 5.28 | 0.025 | 11.47 | 0.001 |
| **Decomposition** | | | | | | | | |
| *Sterile treatment* | *1* | *39* | *2.78* | *0.101* | *2.71* | *0.105* | *2.76* | *0.102* |
| *Species stability residual* | *1* | *39* | *2.25* | *0.140* | *0.99* | *0.324* | *1.95* | *0.169* |
| Species stability overall | 1 | 40 | 4.56 | 0.037 | 3.45 | 0.069 | 4.68 | 0.035 |
| **C accumulation** | | | | | | | | |
| *Sterile treatment* | *1* | *39* | *1.97* | *0.166* | *1.95* | *0.168* | *1.95* | *0.168* |
| *Species stability residual* | *1* | *39* | *0.78* | *0.380* | *<0.01* | *0.955* | *0.07* | *0.785* |
| Species stability overall | 1 | 40 | 2.71 | 0.105 | 0.82 | 0.369 | 1.83 | 0.182 |

**Supplementary file 1 - table 9.** Summary ANOVA results for the effect of the average temporal synchrony in the abundance of individual bacterial and fungal taxa that support a given function on the stability of that ecosystem function after first accounting for the sterile soil treatment. The effect of the sterile soil treatment was fitted ahead of species synchrony and the residual effect of stability are shown in italics. For comparison the overall effect of synchrony prior to accounting for the sterile soil treatment is shown in the grey shaded row below. The bold results highlight where the effect of synchrony remained significant after accounting for the sterile soil treatment. Microbial diversity is the scaled average of the species synchrony of the bacterial and fungal taxa.

|  |  |  | **Bacteria** | | **Fungi** | | **Microbial diversity** | |
| --- | --- | --- | --- | --- | --- | --- | --- | --- |
|  | *DF_num_* | *DF_den_* | *F* | *P* | *F* | *P* | *F* | *P* |
| **Biomass production** | | | | | | | | |
| *Sterile treatment* | *1* | *39* | *30.52* | *<0.001* | *28.18* | *<0.001* | *30.04* | *<0.001* |
| *Species stability residual* | *1* | *39* | ***5.63*** | ***0.021*** | *0.82* | *0.369* | ***4.63*** | ***0.036*** |
| Species stability overall | 1 | 40 | 9.50 | 0.003 | 11.10 | 0.002 | 17.709 | <0.001 |
| **Plant Diversity** | | | | | | | | |
| *Sterile treatment* | *1* | *39* | *16.74* | *<0.001* | *16.50* | *<0.001* | *17.22* | *<0.001* |
| *Species stability residual* | *1* | *39* | *2.05* | *0.157* | *1.19* | *0.280* | ***3.75*** | ***0.058*** |
| Species stability overall | 1 | 40 | 7.11 | 0.010 | 10.45 | 0.002 | 15.86 | <0.001 |
| **Decomposition** | | | | | | | | |
| *Sterile treatment* | *1* | *39* | *2.87* | *0.096* | *2.69* | *0.107* | *2.86* | *0.096* |
| *Species stability residual* | *1* | *39* | ***4.10*** | ***0.048*** | *0.40* | *0.529* | ***4.08*** | ***0.048*** |
| Species stability overall | 1 | 40 | 6.40 | 0.014 | 1.65 | 0.204 | 6.93 | 0.011 |
| **Soil C assimilation** | | | | | | | | |
| *Sterile treatment* | *1* | *39* | *1.98* | *0.165* | *2.03* | *1.95* | *0.168* | *1.95* |
| *Species stability residual* | *1* | *39* | *0.99* | *0.323* | *2.46* | *0.07* | *0.785* | *0.07* |
| Species stability overall | 1 | 40 | 2.13 | 0.150 | 4.42 | 0.040 | 1.83 | 0.182 |
