# Supplementary material for: Diversity and asynchrony in soil microbial communities stabilizes ecosystem functioning
Source: eLife. 2021 Mar 23;10:e62813. doi: 10.7554/eLife.62813 (PMC7987343; doi:10.7554/eLife.62813)
Supplement: Supplementary file 2. [file elife-62813-supp2.docx]

**Supplementary file 2**

Summary results for the taxonomic assignment of OTUs having a significant positive and negative association with an ecosystem function at each time point.

** Supplementary file 2 - figure 1**. The proportion of fungal taxa belonging to a fungal Order that were found to have a significant negative and positive impact on each ecosystem function. Different coloured bars indicate the top eight different fungal Orders that were found to have a significant impact (|SES| > 1.96) on each ecosystem function at each time point. Numbers above indicate the number of fungal taxa from that order with a significant effect.

**Supplementary file 2 - figure 2**. The proportion of bacterial taxa belonging to a bacterial Order that were found to have a significant negative and positive impact on each ecosystem function. Different coloured bars indicate the top eight different fungal Orders that were found to have a significant impact (|SES| > 1.96) on each ecosystem function at each time point. Numbers above indicate the number of bacterial taxa from that order with a significant effect.

**Supplementary file 2 - table 1**. The percentage (%) and number (N) of fungal taxa that had a positive impact on the same ecosystem function at more than one time point. Only the top ten Genera with the most taxa that that had a positive impact on a given function at more than one time point are listed.

|  | Biomass | | Diversity | | Decomposition | | C assimilation | |
| --- | --- | --- | --- | --- | --- | --- | --- | --- |
| Fungal Genera | % | N | % | N | % | N | % | N |
| *Acremonium* | 0 | 0 | 0 | 0 | 3.9 | 1 | 0 | 0 |
| *Apodus* | 0 | 0 | 0 | 0 | 50.0 | 5 | 0 | 0 |
| *Articulospora* | 0 | 0 | 0 | 0 | 10.0 | 2 | 0 | 0 |
| *Cadophora* | 0 | 0 | 0 | 0 | 10.0 | 2 | 20.0 | 7 |
| *Catenulifera* | 0 | 0 | 0 | 0 | 50.0 | 5 | 0 | 0 |
| *Cladophialophora* | 14.8 | 6 | 0 | 0 | 0 | 0 | 7.4 | 2 |
| *Conocybe* | 0 | 0 | 0 | 0 | 0 | 0 | 14.3 | 2 |
| *Cortinarius* | 5.8 | 3 | 5.8 | 3 | 2.6 | 1 | 3.7 | 2 |
| *Cryptococcus* | 4.7 | 3 | 0 | 0 | 0 | 0 | 0 | 0 |
| *Diversispora* | 21.4 | 11 | 0 | 0 | 0 | 0 | 0 | 0 |
| *Entoloma* | 0 | 0 | 5.4 | 3 | 0 | 0 | 0 | 0 |
| *Fusarium* | 0 | 0 | 7.5 | 4 | 0 | 0 | 5.0 | 2 |
| *Glomus* | 3.1 | 3 | 6.2 | 4 | 2.3 | 1 | 3.8 | 2 |
| *Metarhizium* | 0 | 0 | 0 | 0 | 0 | 0 | 20.0 | 5 |
| *Minimedusa* | 0 | 0 | 20.0 | 16 | 13.3 | 3 | 0 | 0 |
| *Mortierella* | 10.0 | 4 | 11.4 | 6 | 3.6 | 1 | 1.4 | 2 |
| *Mucor* | 9.4 | 4 | 12.5 | 8 | 0 | 0 | 6.3 | 2 |
| *Podospora* | 0 | 0 | 14.3 | 11 | 0 | 0 | 0 | 0 |
| *Scolecobasidium* | 0 | 0 | 0 | 0 | 0 | 0 | 6.9 | 2 |
| *Septoglomus* | 0 | 0 | 0 | 0 | 22.2 | 4 | 0 | 0 |
| *Serendipita* | 5.9 | 4 | 5.9 | 4 | 0 | 0 | 0 | 0 |
| *Spizellomyces* | 0 | 0 | 4.3 | 3 | 0 | 0 | 0 | 0 |
| *Tetracladium* | 44.4 | 14 | 0 | 0 | 0 | 0 | 0 | 0 |

**Supplementary file 2 - table 2.** The percentage (%) and number (N) of bacterial taxa that had a positive impact on the same ecosystem function at more than one time point. Only the top ten Orders with the most taxa that that had a positive impact on a given function at more than one time point are listed.

|  | Biomass | | Diversity | | Decomposition | | C assimilation | |
| --- | --- | --- | --- | --- | --- | --- | --- | --- |
| Bacterial Order | % | N | % | N | % | N | % | N |
| Acidimicrobiales | 27.1 | 329 | 0 | 0 | 0 | 0 | 14.0 | 212 |
| Anaerolineales | 19.0 | 157 | 27.3 | 452 | 6.0 | 85 | 0 | 0 |
| Bacillales | 0 | 0 | 0 | 0 | 0.8 | 18 | 0 | 0 |
| Burkholderiales | 0 | 0 | 12.1 | 82 | 0 | 0 | 0 | 0 |
| Clostridiales | 0 | 0 | 0 | 0 | 0 | 0 | 3.4 | 29 |
| Cytophagales | 13.1 | 82 | 0 | 0 | 4.2 | 27 | 0 | 0 |
| Gemmatimonadales | 20.0 | 185 | 26.8 | 253 | 4.6 | 41 | 9.3 | 119 |
| Myxococcales | 12.8 | 72 | 20.4 | 132 | 3.6 | 19 | 6.5 | 38 |
| Planctomycetales | 16.4 | 92 | 23.4 | 223 | 4.3 | 35 | 8.1 | 49 |
| Rhizobiales | 7.1 | 56 | 8.9 | 68 | 1.8 | 18 | 3.0 | 29 |
| Rhodospirillales | 17.4 | 97 | 21.5 | 155 | 5.6 | 53 | 7.0 | 40 |
| Sphingobacteriales | 17.4 | 97 | 15.7 | 107 | 4.2 | 23 | 4.8 | 34 |
| Xanthomonadales | 10.9 | 57 | 15.3 | 89 | 0 | 0 | 5.6 | 36 |
| Unknnown | 15.3 | 138 | 20.9 | 178 | 4.6 | 40 | 7.6 | 63 |
